# Novel Roles of the Picornaviral 3D Polymerase in Viral Pathogenesis

**DOI:** 10.1155/2010/368068

**Published:** 2010-06-29

**Authors:** Jason Kerkvliet, Ramakrishna Edukulla, Moses Rodriguez

**Affiliations:** ^1^Department of Neurology, Mayo Clinic, Rochester, MN 55905, USA; ^2^Department of Immunology, Mayo Clinic, Rochester, MN 55905, USA

## Abstract

The RNA-dependent RNA-polymerase, 3D^pol^, is an essential component in the picornavirus genome for the replication of single stranded RNA. However, transgenic expression of 3D^pol^ in mice has antiviral effects. Here, we discuss the structure and function of 3D^pol^ during picornavirus replication, we review the evidence and consequence of a host immune response to epitopes in 3D^pol^ after picornavirus infection, highlight data showing the antiviral effects of transgenic 3D^pol^ from Theiler's murine encephalomyelitis virus (TMEV), and discuss potential mechanisms by which 3D^pol^ is causing this antiviral effect in mice.

## 1. Introduction

Picornaviruses are members of a large family of viruses which are classified into twelve different genera. These viruses can cause acute illness as benign as the common cold to chronic illness like poliomyelitis in humans, and foot and mouth disease in split-hoofed animals. Picornavirus was derived from the word “pico” meaning small and “rna” for the single stranded ribonucleic acid it contains. The RNA genomes of picornaviruses are positive sense and can range between 6.7 to 9.5 kilobases. Picornaviral virions are nonenveloped, however, the RNA genome is encapsulated in an icosahedral protein structure made from four capsid proteins encoded by the virus. 

**Figure 1:**
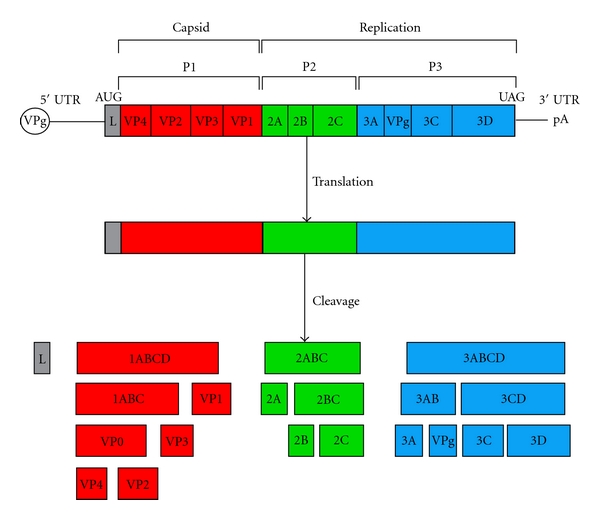
Genomic organization of picornaviruses with the covalently linked VPg peptide as it is packaged in the virion. The regions of the P1 (pink), P2 (blue), and P3 (brown) are shown along with the viral proteins encoded within each domain. P1 encodes capsid proteins and the P2 and P3 domains encode noncapsid proteins used for protein processing and replication. The plus-sense single stranded RNA genome is translated into a single polyprotein and then cleaved by cis and transacting viral proteases.

The single positive-stranded RNA genome of the virus is translated into a single polyprotein which is divided into three regions P1, P2, and P3 (Figure 1). P1 encodes proteins that make up the viral capsid (consisting of VP1, VP2, VP3, and VP4), whereas the P2 and P3 regions encode proteins that are involved in protein processing and genome replication. Cleavage of the polyprotein is carried out by proteinase 2A^pro^, 3C^pro^, or 3CD^pro^. 3CD^pro^ can then be cleaved further into 3C^pro^ and 3D^pol^. The fourth and final protein domain of the P3 region is 3D^pol^, an RNA-dependent RNA-polymerase which drives replication of both plus and minus stranded viral RNA and is the main focus of this paper. 

3D^pol^ was first characterized from experiments which used cell lysates from polio-infected cells to denote RNA polymerase activity using radioactive nucleotide incorporation into viral RNA [1]. 3D^pol^ associates with cellular membrane fractions in a complex of viral proteins that forms the RNA replication complex in which 3D^pol^ is the major component [2]. 3D^pol^ later was found to be a template and primer dependent enzyme, when recombinant 3D^pol^ purified from bacteria could copy poliovirus RNA in a cell-free system only in the presence of an oligo (U) primer [3, 4]. 

 This paper will focus on RNA-dependent RNA-polymerase, 3D^pol^, and recently published data which shows that transgenically expressed 3D^pol^ from Theiler's murine encephalomyelitis virus (TMEV) inhibits viral replication and subsequent infection [5]. This effect appears to be broader than inhibiting TMEV as we will show data of the antiviral effect of 3D using another picornavirus and a nonpicornavirus infection in mice. We will first review the sequence and structural homologies of the 3D polymerase between different viruses in the picornavirus family. Secondly, we will review host immune recognition of 3D in mice and contrast this to other picornavirus proteins. Finally, we will review our recently published data and new data showing the antiviral effect of transgenic 3D and discuss potential mechanisms of how 3D polymerase is causing this antiviral effect.

## 2. Structure and Function of 3D^**pol**^

**Figure 2:**
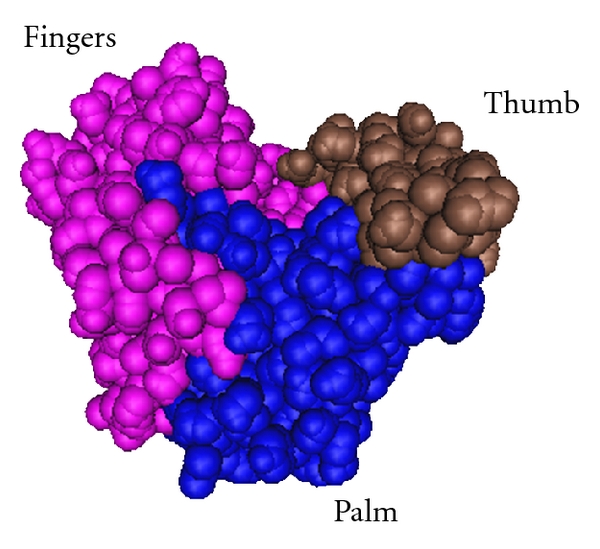
Crystal structure of 3D polymerase from FMDV in the “right-handed” configuration typical of polymerases showing fingers (red), palm (green), and thumb (blue) domains [8]. Structure was obtained from the molecular modeling database (MMDB ID: 29094) at the NCBI web site and illustrated using Cn3D [34, 35].

Although the overall amino acid sequences of 3D polymerase between different picornaviruses are not very homologous, the basic overall structure and binding motifs between them are very similar (Table 1). Crystal structures from poliovirus, rhinovirus, and foot and mouth disease virus polymerases showed resolution of all amino acids in 3D^pol^ and showed that the enzyme has the same overall structure as other DNA and RNA polymerases in that it contains finger, palm, and thumb domains [8–11] (Figure 2). Four motifs in the palm are identical to all other RNA polymerases, and the fifth domain is similar to other RNA dependent, but not DNA dependent polymerases [9, 12, 13]. Furthermore, the amino acid sequence residing in these motifs are highly conserved between different picornaviruses [14]. The amino acids at the N-terminus of 3D polymerase were found to be important in the function of this enzyme. The N-termunis encircles the active site in the palm domain and mutations in the N-termunis have been shown to disrupt 3D polymerase activity [8, 11, 15]. More recently, a coxsackievirus 3D polymerase demonstrated the importance of hydrophobicity at residue 5 where the polymerase was more active with amino acids which were more hydrophobic at this residue [16]. This is important for stabilizing the structure of 3D during a conformational change that occurs at the nucleotide repositioning step. 

In 1995, efficient polymerization by poliovirus 3D polymerase was found to be concentration dependent, and chemical cross-linking of proteins after initiation of elongation found many polymerase-polymerase interactions [17]. Later the first crystal structure of 3D polymerase found many intermolecular interactions important in polymerase function [9]. The polymerase has two interfaces which allows each molecule to form a head to tail configuration and oligomerize [9]. Interface I involves interactions with residues on the back side of the thumb with residues on the back of the palm to align each molecule head to tail to form long fibers. Interface II involves intermolecular donation of the NH2-terminus from one molecule to the thumb region of another polymerase molecule forming another head to tail oriented fiber strand. The fibers formed from both interface I and interface II interactions intersect each other at a 90-degree angle to form a two-dimensional array. This 3D polymerase lattice was visualized on membranous vesicles in poliovirus-infected cells by electron microscopy [18]. However, the function of this lattice is not known, nor is it known whether this same lattice occurs in all picornaviruses expressing 3D polymerase. A virus containing mutations to amino acids Arg-455 and Arg-456 in the back of the thumb disrupted the formation of polymerase fibers and the virus was not viable, however, the lack of viral replication from these mutations was the result of a disruption in the interaction of 3D with the 3C domain. Furthermore, mutations to residues Asp-339, Ser-341, and Asp-349 in the palm domain also disrupted the formation of fibers, and despite reduced replication the virus remained viable. This indicated that the formation of fibers along interface I is not essential for virus viability [19]. Although these intermolecular interactions may not be essential for viral replication and viability, they are important for efficient replication of virus as discovered by biochemical assays using mutated polymerases [20]. 

3D polymerase not only interacts with itself but also forms molecular interactions with other viral molecules during genomic replication. The chronological sequence of steps and the molecular interaction that occurs during viral replication is not completely understood and varies slightly among different picornaviruses. However, all picornaviruses have the peptide, VPg, covalently linked to the 5′ end of their genome. VPg is a 22 amino acid peptide derived from the 3B portion of 3AB and serves as a primer for 3D^pol^ after VPg is uridylylated to form VPg-pUpU. The process of VPg uridylylation and initiation of replication of 3D^pol^ takes place in a complex of viral RNA proteins that reside on membranous vesicles called the RNA replication complex. The RNA replication complex in picornaviruses sequester viral replication components in a localized area to increase the efficiency of RNA replication [21]. Many studies have determined what viral and host proteins are involved in VPg uridylylation and initiation of RNA replication. In this replication complex, 3AB resides in the lipid bilayer of the membranous vesicles. Fluorescent membrane topography studies in model membrane vesicles showed that membrane-bound 3AB is highly efficient in stimulating 3D polymerase activity, in contrast to 3A alone [21]. This same study showed that the release of VPg by cleavage of 3AB with proteinase 3CD could be uridylylated. Residues important in the interaction and recruitment of the 3D subunit to 3AB take place through the binding of the B subunit (VPg) in 3AB and the base of the thumb in the 3D subunit [22, 23]. This interaction recruits 3CD dimers and/or 3D itself, as 3CD dimers are important to the recruitment of mature 3D polymerase to the replication complex through interactions with a region of 3C and amino acids in the back of the thumb in 3D polymerase [24, 25]. The “sea” of 3AB molecules in the membrane serves as the source of VPg after the membranous 3AB is cleaved by the recruited 3CD protease [26]. 3AB is not the only precursor protein that may be a donor for VPg. A mutant poliovirus which introduced a defective cleavage site between 3B and 3C, replicated and produced 3BC-linked RNA [27, 28]. Also, an in vitro uridylylation assay using a 15-nucleotide RNA template and poliovirus polymerase revealed that 3D can uridylylate other 3D molecules as well as viral 3CD and 3AB precursors [29]. After being cleaved, VPg interacts with 3D polymerase through residues in the pocket of the palm [8, 30]. VPg bound to 3D^pol^ is then uridylylated using a cis acting replication element located in the open reading frame of picornaviruses as a template via a “slide back" mechanism [31]. After the uridylylation of VPg, VPg-pUpU translocates to the 3′ end of negative sense strands where it serves as a primer for 3D polymerization in the initiation of positive stranded RNA synthesis. Negative stranded synthesis is initiated by a different mechanism as mutations to cre that block Vpg uridylylation at this site do not effect synthesis of negative stranded RNA [32, 33].

## 3. Host Immune Response to 3D^**pol**^

Until recently, T and B cell-specific responses to 3D polymerase in picornaviral infections had not been characterized. Numerous studies have been done to elicit which viral peptides are recognized by the adaptive immune system, however, most of the peptides important in viral clearance were found to be capsid proteins. Although there are many picornaviruses which may elicit a 3D specific immune response, we will focus on the TO subgroup of TMEV because immune responses to this subgroup has been studied in depth as cerebral infection with this subgroup of TMEV is used in mouse models for demyelinating diseases such a multiple sclerosis in mouse strains that are susceptible to a persistent viral infection in the spinal cord. There are also mice resistant to persistent TMEV infection. Resistant mice have similar acute viral encephalitis in the brain after intracerebral infection, however, the virus is then cleared from the CNS. Using susceptible and resistant mouse strains the genetic factors associated with viral susceptibility map to the H-2D locus of the class I major histocompatability complex [36–38]. Mice containing the H-2^b,d,k^ haplotypes are resistant to persistent infection whereas H-2^f,p,q,r,s,v^ haplotypes are susceptible. Clearance of the virus from the CNS in resistant haplotypes is dependent on a class I restricted CD8^+^ cytotoxic T lymphocyte (CTL) response, because normally resistant mice that are depleted of CD8^+^ T cells or lack the expression of beta 2 microglobulin (b2M) become susceptible to persistent viral infection [39–41]. 

 After it was determined that a class I restricted CTL response was important for viral clearance of TMEV in mice, studies were done to find the viral antigens which were recognized by CTL's. It was shown that capsid proteins expressed transgenically in mice with a genetically resistant background became susceptible to persistent TMEV infection, in contrast to mice that expressed noncapsid proteins such as the 3D polymerase [42]. Later it was found that the peptide VP2_121–130_ was the immunodominant peptide conferring resistance in mice expressing H-2D^b^ [43]. 

Although class I restricted CTL responses to viral capsid antigens determines whether mice clear TMEV, class II responses to viral antigens also occur and play a role in CNS injury in the absence of a strong CTL response [44]. Recently SJL mice were found to have a dominant CD4^+^ T-cell response to a peptide epitope in 3D polymerase [45]. 3D peptide-specific CD4^+^ T cells were found in both SJL and B6 mice after infection with TMEV, with the susceptible SJL mice containing more 3D specific T cells than the resistant B6 mice. Furthermore, it was determined through preimmunization and tolerization of mice, that CD4^+^ T cells specific to the peptide epitope 3D_21–36_ play a more pathogenic role in susceptible SJL mice than CD4^+^ T cells specific to the capsid epitopes (VP1_233–250_, VP2_74–86_, VP3_24–37_), because deletion of 3D_21–36_ peptide specific T cells reduced demyelination and clinical scores in SJL mice. 

Of interest, CD4^+^ T cells can be pathogenic or protective depending on the viral epitope. The most important factor in viral clearance is restricted to strong CD8^+^ CTL response to viral capsid antigens. However, in the absence of a strong CTL response the viral antigen for which the majority of CD4^+^ T cells are specific to may have a major influence on the severity of disease in a persistent viral infection. Because the available T-cell repertoire in the periphery is dependent on the mouse haplotype, mice with susceptible haplotypes may have different numbers of 3D_21–36_ specific CD4^+^ T cells in there repertoire, contributing to different degrees of injury. It is not known why 3D_21–36_ specific T cells are more pathogenic, but it could be that 3D_21–36_ CD4^+^ T cells are only weakly stimulated by these epitopes. This would then lead to weak recruitment of other immune cells to the area of infection resulting in nonclearance and ongoing persistent infection that smolders and damages nearby cells in the CNS.

## 4. 3D as an Antiviral Molecule

Since 3D polymerase plays such a central role in picornavirus replication many strategies have been devised to inhibit the function of 3D and ultimately slow the replication of picornaviruses. One such strategy is the use of nucleoside analogs, such as ribavirin which increases the error rate of the already low fidelity 3D polymerase into a state of lethal mutagenesis. However, there are frequent side effects because most nucleoside analogs also incorporate into host cell RNA. Also, the virus becomes resistant to treatment. The idea that 3D polymerase is a target for antiviral therapy is logical because proper function of 3D is required for picornaviral replication. However, transgenic expression of 3D having antiviral properties is not as easily explained. Our published data is the first using transgenic 3D mice to show antiviral effects. We originally made 3D transgenic mice to be used as a control transgene to study the effects of tolerance to other picornaviral proteins (VP1, VP2, VP3, etc.). At that time, 3D was not thought to be an important antigen for T cells involved in viral clearance. However, after quantifying viral transcripts in 3D transgenic mice we noticed these mice had 100 to 1000 fold reductions in viral RNA after intracranial infection with TMEV compared to nontransgenic mice or transgenic mice expressing other viral capsid proteins. For this reason, the mechanism behind the antiviral effect of transgenic 3D may produce an “outside the box" explanation that will open new avenues for the way scientists think about antiviral research. 

**Figure 3:**
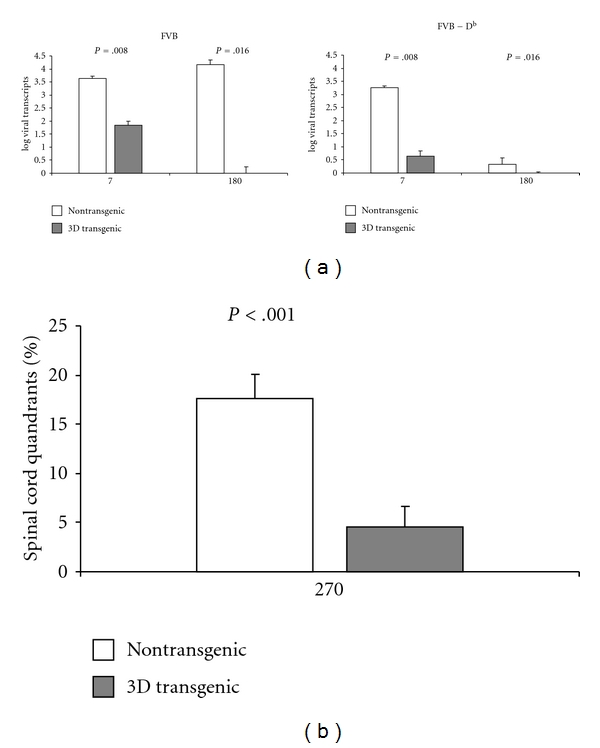
Viral transcripts and demyelination in mice infected with TMEV. (a) Nontransgenic and 3D transgenic mice were infected with 2 × 10^5^ plaque forming units of TMEV intracerebrally. Brain and spinal cord were harvested after 7 and 180 days and total RNA was isolated using Trizol reagent (Invitrogen). Viral transcripts for TMEV were quantified by real-time RT-PCR using primers specific for the VP2 region of TMEV. Data are expressed as mean relative viral transcripts + SEM (*n* = 5 for nontransgenic and 5 for 3D transgenic) in the brain or spinal cord above uninfected mice and were normalized to GAPDH. (b) Percent of spinal cord quadrants containing demyelinated lesions in 3D transgenic and nontransgenic FVB mice at 270 days after infection. Statistical significance was determined using the Mann-Whitney rank sum test.

FVB mice were made transgenic by microinjection of a linearized vector with the coding region of 3D polymerase from the DA strain of TMEV containing a C-terminus histidine tag. A human ubiquitin promoter was placed upstream of the 3D gene for constitutive expression of 3D in all tissues. We demonstrated that 3D transcripts were present in all tissues examined. The amount of 3D transcripts varied in each tissue with the CNS showing the highest 3D transcript levels. After intracranial infection with TMEV, viral infection was inhibited in these mice compared to infected nontransgenic FVB mice and the virus was cleared after the acute phase of the infection. This led to decreased brain and spinal cord pathology and consequently the preservation of neurons and function in mice containing the 3D transgene. Transgenic mice expressing 3D had less viral transcripts in the spinal cord at both 7 and 180 days after infection with TMEV than nontransgenic control mice (Figure 3(a)). Because of the antiviral effects of the 3D transgene these mice controlled the virus by 180 days as no detectable viral transcripts were present at this time point whereas the virus persisted in the spinal cord of nontransgenic mice. This reduction in viral load caused less demyelination in 3D transgenic mice at 270 days postinfection (Figure 3(b)). 

 After studying the effects of 3D in FVB mice that are susceptible to persistent TMEV infection, we crossed them to FVB mice that contained the H-2D^b^ transgene (FVB-D^b^ mice). T cells in FVB-D^b^ mice respond better to TMEV infection and clear the virus making these mice resistant to persistent infection of the spinal cord. Because these mice have a different T-cell repertoire, containing H-2D^b^ specific T cells, than the wild-type FVB mice which contain a T-cell repertoire that only recognizes the H-2q class I molecules, we could test whether the 3D transgene would further inhibit infection in mice of a different haplotype. If the 3D transgene failed to have an antiviral effect in FVB-D^b^ mice it would have suggested that transgenic 3D expression may be acting through the adaptive immune system because FVB-D^b^ mice already efficiently clear the virus, therefore the effect of 3D would be lost in the background. However FVB-D^b^ mice containing the 3D transgene showed decreased viral loads during the acute phase of the infection. Viral transcripts in the spinal cord were significantly reduced in FVB-D^b^ mice containing the 3D transgene versus nontransgenic FVB-D^b^ mice at 7 days (Figure 3(a)). These observations led us to hypothesize that transgenic 3D is not altering the adaptive immune response to more effectively clear the virus for the following reasons. First, a significant viral inhibitory effect was seen in the spinal cord at the same time as the primary adaptive immune response to the virus had peaked (7 days postinfection). If this reduction in viral load was caused by a difference in adaptive immunity, then we would not have expected to see a significant difference in viral loads until a few days after the primary adaptive immune response had peaked. Second, the effect of 3D polymerase on viral infection was present in FVB-D^b^ mice despite their more efficient viral immune system. To further test the theory that 3D was not working via the adaptive immune response, we crossed the 3D transgenic mice to FVB mice that had the Rag 1 gene knocked out (FVB-Rag^−/−^). These mice are completely void of T and B cells. FVB-Rag^−/−^ mice containing the 3D transgene had 10-fold less virus 14 days after infection with TMEV and lived longer than nontransgenic FVB-Rag^−/−^ mice. Although, we do not know whether a change in the adaptive immune system provides an additive effect to support what we saw in 3D transgenic mice, this data strongly indicates that the adaptive immune system was not the primary reason for the viral inhibition by transgenic 3D polymerase. 

 It is known that the random integration of transgenes could affect genes adjacent to the insertion site by disrupting the coding frame of a gene or disrupting regulatory sequences of a gene(s). Pathology data at the 45-day-time point showed that a second 3D transgenic mouse line had even more significant reduction in spinal cord pathology than the original line, which ruled out any “insertional effect" from the random integration of the transgene itself. 

**Figure 4:**
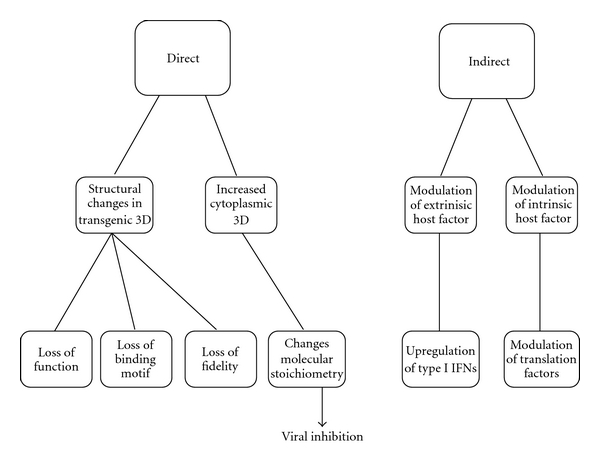
Flowchart demonstrating the potential mechanisms of 3D^pol^ antiviral effect on viruses.

**Figure 5:**
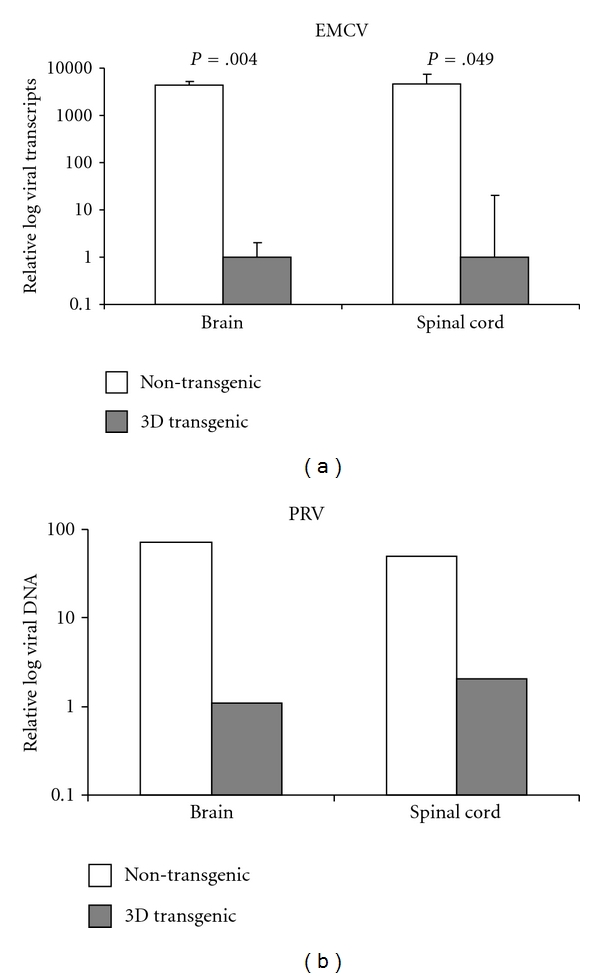
Viral load in mice infected with EMCV or PRV. (a) Viral transcripts in EMCV infected mice. Nontransgenic and 3D transgenic FVB mice were infected with 40 plaque forming units of EMCV (VR-129B ATCC) intraperitoneally. Brain and spinal cord were harvested after 3 days and total RNA was isolated using Trizol reagent (Invitrogen). Viral transcripts for EMCV were quantified by real-time PCR using primers specific for the VP2 region of EMCV. Data were normalized to GAPDH transcripts and are expressed as mean relative viral transcripts + SEM (*n* = 3 for nontransgenic and 4 for 3D transgenic). Data was statistically significant using a two-tailed student's *t*-test assuming unequal variance. (b) Viral DNA in pseudorabies virus (PRV) infected mice. Nontransgenic and 3D transgenic FVB mice were infected with 2 × 10^7^ plaque forming units of PRV 152 intramuscularly in the left hind limb. Brain and spinal cord were harvested after 7 days and DNA was isolated. Viral DNA for PRV was quantified by real-time PCR using primers specific for the inserted EGFP in PRV 152. Data are expressed as mean relative viral DNA (*n* = 2 for nontransgenic and 3 for 3D transgenic) and were normalized to genomic IL-2 DNA.

 To understand the possible mechanisms for which 3D may be working we developed a flow diagram as to the possible places that 3D may function as an antiviral protein (Figure 4). Transgenic 3D may interact directly through the virus itself as it enters the cell. As mentioned previously, 3D oligomerizes with itself and can form a lattice of 3D molecules. 3D also binds and interacts with many other viral proteins and RNA. Transgenic 3D may be processed differently in the host cell disrupting one or more binding motifs that are important in interacting with other viral molecules. Therefore, transgenic 3D could act as a dominant negative form of the viral 3D thus competing for or interfering with binding sites. The function or folding structure of the transgenic 3D molecule has not been studied making this hypothesis plausible. Also, new data from viral transcripts measured in mice infected with EMCV, another picornavirus very similar to TMEV, showed similar viral inhibitory effects, which provided more evidence to support this hypothesis. After infection of both 3D transgenic and nontransgenic wild-type mice with 40 PFU of EMCV intraperitoneally, 3D transgenic mice had 1000 fold less viral transcripts in the brain and spinal cord just 3 days after infection (Figure 5(a)). However, evidence against a direct dominant negative effect of 3D was found later. Data using a nonpicornavirus indicate that 3D also has an antiviral effect against viruses other than picornaviruses. Because of its ability to infect neurons, we infected mice with the pseudorabies virus (PRV). PRV is a dsDNA alphaherpes virus that is tropic to axon terminals and is transported retrogradely up the neuron to the CNS. The virion does not encode an RNA-dependent RNA-polymerase like 3D in its genome. After infection with 2 × 10^7^ PFU of PRV intramuscularly in the hind limb, mice containing the 3D transgene had 100 fold less virus 6 days after infection (Figure 5(b)). This indicated that transgenic 3D polymerase has antiviral properties on a broad spectrum of viruses and argues strongly against a dominant negative effect as the mechanism through which 3D functions as an antiviral. Furthermore, stably transfected cell lines, thus far, have failed to reproduce the effect seen in transgenic mice. Even if the function and binding properties of the transgenic 3D were no different from viral 3D polymerase, a change in the stoichiometry of the molecules in the cytoplasm may decrease the rate of viral replication in picornaviruses. Studies have shown that the stimulatory effect of 3AB on 3D polymerization is inhibited with increasing concentrations of 3D polymerase in vitro [46–48]. However, this hypothesis suffers the same arguments previously mentioned. Because of the finding that transgenic 3D inhibits a dsDNA virus which does not encode an RNA-dependent RNA-polymerase, we have considered indirect mechanisms through which transgenic 3D may be functioning. Possible indirect mechanisms could be that transgenic 3D is affecting viral replication either by regulating cell extrinsic factors such as Type I IFNs, cell intrinsic factors such as host translational initiation factors, or host RNA-binding factors that interact with viral proteins or nucleotides. The expression of secreted proteins that effect the replication of the virus or innate immune responses may be upregulated by 3D. Type I IFN's are early response cytokines of the innate immune system which are important in controlling many viral infections [49, 50]. Up-regulation of Type I IFN's is triggered by the presence of dsRNA in virally-infected cells. Therefore, we hypothesize that the presence of functional transgenic 3D molecules in the cytoplasm as the virus enters may allow dsRNA synthesis to occur sooner, in contrast to viral replication without transgenic 3D where transcription and processing of all viral proteins in the viral genome has to occur before RNA synthesis can take place. Earlier dsRNA synthesis may theoretically lead to an accumulation of dsRNA molecules in the cytoplasm which could then bind the appropriate receptor to initiate transcription of type I IFN in infected cells sooner when transgenic 3D is present. However, the limitations to this are that many other viral proteins may need to be transcribed and processed to form a replication complex to allow efficient RNA synthesis by transgenic 3D. Another way transgenic 3D could up-regulate host proteins is by replicating host mRNA transcripts which encode these proteins. Previous studies showed that host transcription is shut off through translocation of proteolytic 3CD^pro^ to the nucleus via a nuclear localization sequence located near the N-terminus of the poliovirus 3D polymerase [51, 52]. Therefore, transgenic 3D itself may translocate to the nuclease and theoretically replicate gene transcripts if the right secondary structure is presented. Limitations of the hypothesis for indirect mechanisms is the lack of scientific literature to support the specific molecules needed for this function. Therefore further investigation will be needed to test this hypothesis. However, knowing that the antiviral effect seen in 3D transgenic mice occurs in both DNA and RNA viruses, it is possible that this unexpected observation will uncover new and interesting strategies for antiviral research.

##  Disclosures 

The authors have nothing to disclose.

## Figures and Tables

**Table 1:** Amino acid sequence alignment of 3D^pol^ from five different picornaviruses to TMEV 3D^pol^ (Swiss Prot Accession: P13899) which was used for the query sequence. The sequence alignment was run using Blastp from National Center for Biotechnology Information (NCBI) web site for 2 or more sequences [6, 7]. Column heading definitions: Accession = the accession # from the Swiss Prot database of the aligned sequence. Identities = percentage of amino acids that are identical to the query in the alignment. Positives = the percentage of amino acid that have similar physical properties to the query in the alignment. Gaps = percentage of amino acids that create a gap to the query in the alignment. Score = Bit score. This is derived from the raw alignment score in which the statistical properties of the scoring system used have been taken into account. Because bit scores have been normalized with respect to the scoring system, they are used to compare alignment scores from different searches. *E* value = Expectation value. The number of different alignments with scores equivalent to or better than this alignment that are expected to occur in a database search by chance. Hence, the lower the *E* value, the more significant the score.

	Accession	Identities	Positives	Gaps	Score (Bits)	*E* value
EMCV	P3304	65	78	0	627	0.*E* + 00
FMDV	P3305	41	62	2	370	3.*E* − 106
HRV	P3303	32	53	5	237	4.*E* − 66
PV	P3300	31	52	6	234	2.*E* − 65
EV71	Q66478	29	50	4	207	4.*E* − 57
